# Association Between Long-Term Use of Non-steroidal Anti-inflammatory Drugs and Hyperkalemia in Diabetic Patients

**DOI:** 10.7759/cureus.15648

**Published:** 2021-06-14

**Authors:** FNU Sahil, Jatender Kumar, Gul Raiz, Naila S Bhutto, Hamza Tahir, Zauraiz Anjum, Sidra Naz, Amber Rizwan, Maha Jahangir, Sania Muhammad Shehzad

**Affiliations:** 1 Internal Medicine, Shaheed Mohtarma Benazir Bhutto Medical University, Larkana, PAK; 2 Internal Medicine, Jinnah Postgraduate Medical Center, Karachi, PAK; 3 Internal Medicine, Akhtar Saeed Medical and Dental College, Jhang, PAK; 4 Internal Medicine, Chandka Medical College, Chandka, PAK; 5 Internal Medicine, Allama Iqbal Medical College, Lahore, PAK; 6 Internal Medicine, Fatima Jinnah Medical University, Lahore, PAK; 7 Internal Medicine, University of Health Sciences, Lahore, PAK; 8 Family Medicine, Jinnah Postgraduate Medical Center, Karachi, PAK; 9 Anesthesiology, Dow University of Health Sciences, Karachi, PAK

**Keywords:** drug induced hyperkalemia, nsaids, diabetes type 2, association, adverse event

## Abstract

Introduction

The association between continuous use of nonsteroidal anti-inflammatory drugs (NSAIDs) and hyperkalemia is not consistent in the literature and creates grounds for further large-scale trials, particularly in patients with a chronic disease that affects renal function, such as diabetes mellitus (DM). In this study, we will compare mean serum potassium level and the prevalence of hyperkalemia in diabetic and non-diabetic patients based on their use of NSAIDs.

Methods

This case-control study was conducted in the internal medicine unit of a tertiary care hospital from May 2019 to December 2020. After taking informed consent, 700 patients with a confirmed diagnosis of type 2 DM, of either gender, were enrolled in the study via consecutive convenient non-probability technique. Another set of 700 participants from the public were enrolled as the reference or control group. Continuous NSAID use was defined as NSAID used for a minimum of 20 days in the last 30 days. Blood was drawn via phlebotomy and sent to the laboratory to test for potassium level.

Results

Serum potassium level was significantly higher in diabetic patients with continuous NSAID use compared to the diabetic patients without continuous use (4.8 ± 0.8 mmol/L vs. 4.5 ± 0.7 mmol/L; p-value: 0.0001). Additionally, serum potassium level was significantly higher in non-diabetic patients with continuous NSAID use compared to non-diabetic patients without continuous use (4.3 ± 0.7 mmol/L vs. 3.9 ± 0.5 mmol/L; p-value: 0.0001)

Conclusion

In this study, the patients with continuous use of NSAIDs had higher levels of serum potassium level compared to patients without continuous use of NSAIDs. This difference was even higher in diabetic patients.

## Introduction

Hyperkalemia is a potentially life-threatening metabolic problem, defined as a serum potassium concentration of greater than 5.0 mmol/L. Drug-induced hyperkalemia is the most common cause of raised potassium levels in day-to-day clinical practice [1]. Drug-induced hyperkalemia usually occurs in patients with an underlying renal disorder or other factors interfering with potassium homeostasis [2]. The mechanism for drug-induced hyperkalemia involves impaired renal potassium excretion, transcellular potassium shift, or increased potassium supply [2].

Nonsteroidal anti-inflammatory drugs (NSAIDs), commonly used for pain or inflammation, have been a well-known drug for causing acute kidney injury (AKI) and secondary hyperkalemia. Many clinical guidelines have warned about its use in patients with chronic kidney disease (CKD), other comorbidities, and in older adults. A large population-based cohort study of older adults in 2019 concluded that receiving a new NSAID prescription for more than 14 days is associated with a higher 30-day risk of AKI and hyperkalemia compared with a non-NSAID user [3]. In contrast, a recent Canadian population-based cohort study of older adults with CKD, congestive heart failure, diabetes mellitus (DM), and hypertension found no significant association between newly prescribed NSAIDs use and hospitalization with AKI or hyperkalemia [4]. Similarly, two case-control studies have shown contradictory reports related to the association between hyperkalemia and NSAIDs [5,6]. Thus, the association between NSAIDs and hyperkalemia is not consistent in the literature and creates grounds for more large-scale trials, particularly in patients with a chronic disease that affects renal function such as DM. In this study, we compared mean serum potassium level and the prevalence of hyperkalemia in diabetic and non-diabetic patients based on their use of NSAIDs. The result of this study will assist physicians in being more judicious in the use of NSAIDs in diabetic patients.

## Materials and methods

This case-control study was conducted in the internal medicine unit of a tertiary care hospital from May 2019 to December 2020. After taking informed consent, 700 participants with a confirmed diagnosis of type 2 DM, of either gender, were enrolled in the study via consecutive convenient non-probability technique. These participants were already on treatment for their diabetes. Another set of 700 participants coming to the outpatient department (OPD) without type 2 DM were enrolled as the reference or control group. Patients with chronic kidney disease were excluded from the study. Similarly, patients on diuretics and spironolactone were not included in the study.

After enrollment, the patient’s history including age, gender, smoking status, comorbidities, and use of NSAIDs was noted in a self-structure questionnaire. Continuous NSAID use was defined as NSAID used for a minimum of 20 days (minimum of two tablets per day) in the last 30 days. Blood was drawn via phlebotomy and sent to the laboratory to test for potassium level. A potassium level greater than 5.0 mmol/L was labeled as hyperkalemia [1].

Statistical analysis was done using the SPSS Statistics version 23.0 (IBM Corporation, Armonk, New York, United States). Categorical data were presented as frequency and percentage. Numerical data were presented as mean and standard deviation. T-test and Chi-square were applied as appropriate. A p-value of less than 0.05 meant that the difference between the groups is significant and the null hypothesis is void.

## Results

There was no difference in demographics and risk factor profile between the two groups (Table 1).

**Table 1:** Characteristics of the study participants NSAID: nonsteroidal anti-inflammatory drug; NS: non-significant

Characteristics	Diabetic group (n=700)	Non-diabetic group (n=700)	p-value
Age in years (Mean ± SD)	53 ± 10	54 ± 10	NS
Male (%)	371 (53.0%)	359 (51.2%)	NS
Smoker	214 (30.5%)	251 (35.8%)	NS
Hypertensive	241 (34.4%)	222 (31.7%)	NS
Continuous NSAID use	112 (16.0%)	141 (20.1%)	NS

Serum potassium level was significantly higher in diabetic patients with continuous NSAID use compared to the diabetic patients without continuous use (4.8 ± 0.8 mmol/L vs. 4.5 ± 0.7 mmol/L; p-value: 0.0001). Additionally, serum potassium level was significantly higher in non-diabetic patients with continuous NSAID use compared to non-diabetic patients without continuous use (4.3 ± 0.7 mmol/L vs. 3.9 ± 0.5 mmol/L; p-value: 0.0001) (Table 2).

**Table 2:** Comparison of the effect of NSAID use on serum potassium level in the diabetic and non-diabetic groups A: T-test applied to the diabetic group with and without continuous NSAIDs use B: T-test applied to the non-diabetic group with and without continuous NSAIDs use C: T-test applied to the diabetic and non-diabetic group with continuous NSAIDs use D: T-test applied to the diabetic and non-diabetic group without continuous NSAIDs use NSAID: nonsteroidal anti-inflammatory drug; mmol/L: millimole per liter

Continuous NSAID use	Serum potassium level (mmol/L)		p-value ^A^	p-value ^B^	p-value ^C^	p-value ^D^
	Diabetic group	Non-diabetic group				
Yes	4.8 ± 0.8	4.3 ± 0.7	0.0001	< 0.0001	< 0.0001	< 0.0001
No	4.5 ± 0.7	3.9 ± 0.5				

Prevalence of hyperkalemia was significantly higher in diabetic patients with NSAID use compared to diabetic patients without continuous NSAID use (36.6% vs. 13.7%; p-value < 0.00001). Additionally, the prevalence of hyperkalemia was significantly higher in the non-diabetic patients with continuous NSAIDs use compared to non-diabetic patients without continuous use (19.1% vs. 5.0%; p-value: < 0.0001). Prevalence of hyperkalemia was higher in diabetic patients with continuous NSAID use compared to the non-diabetic patients with continuous NSAID use (36.6% vs. 19.1%; p-value < 0.00001) (Table 3).

**Table 3:** Comparison of the effect of NSAID use on prevalence of hyperkalemia in the diabetic and non-diabetic groups A: T-test applied to the diabetic group with and without continuous NSAID use B: T-test applied to the non-diabetic group with and without continuous NSAID use C: T-test applied to the diabetic and non-diabetic group with continuous NSAID use D: T-test applied to the diabetic and non-diabetic group without continuous NSAID use NSAID: nonsteroidal anti-inflammatory drug

Continuous NSAID use	Hyperkalemia		p-value ^A^	p-value ^B^	p-value ^C^	p-value ^D^
	Diabetic group (n=700)	Non-diabetic group				
Yes	41 (36.6%)	27 (19.1%)	< 0.00001	< 0.00001	< 0.00001	< 0.00001
No	81 (13.7%)	28 (5.0%)				

## Discussion

Our study demonstrates that elevated potassium levels were found in diabetic patients who used NSAIDs for more than 20 days. Similarly, the non-diabetic group using NSAIDs also showed raised potassium levels. However, a comparison between the potassium levels after continuous use of NSAIDs of the diabetic and non-diabetic groups suggested that hyperkalemia was significantly more prevalent in diabetics.

NSAID-induced hyperkalemia is usually due to several pre-existing comorbidities and coming in contact with medicines that can disturb the renal function of balancing potassium. However, there is a scarcity of literature suggesting NSAIDs alone as the only cause of hyperkalemia [6]. Lafrance et al. conducted a nested case-control study which demonstrated that the risk of developing hyperkalemia >6.0 mEq/L only by the administration of NSAIDs in a veteran patient population showed no increase, regardless of the usage being single or multiple times [6]. However, some agents, namely rofecoxib (odds ratio [OR], 1.37) and indomethacin (OR, 1.36), were observed to cause a spike in the risk, not related to cycloxygenase-2 sensitivity [6]. The most common risk factors for the development of hyperkalemia include a history of hyperkalemia, being hospitalized in the previous month, DM, and AKI [6].

Since NSAIDs are known to cause cardiovascular, renal, and gastrointestinal risks [7], these should be kept in mind when dealing with DM, since they are at a greater risk. American Family Physicians have advised all patients with hypertension, heart diseases, CKD of all sorts, and DM to avoid the use of NSAIDs [8]. According to the Canadian guidelines for managing sick days, NSAIDs can lessen the renal blood flow, making patients prone to dehydration [9]. In that case, its use should be discontinued for quite some time. Moreover, complications have been observed by short as well as long-term use [10-12].

Prostaglandins are known to regulate renal potassium; therefore, the inhibition of prostaglandins leads to NSAID-linked hyperkalemia. The release of renin due to prostaglandin, in turn, causes potassium excretion in the distal tubule under the influence of aldosterone. The deficiency of prostaglandin induces hyperaldosteronism, which would potentially cause decreased secretion of potassium. This is the basic phenomenon of NSAID-triggered hyperkalemia [13]. When less sodium chloride is supplied to the distal nephron, the electrochemical gradient for potassium excretion is also disturbed [13], meanwhile, the less active potassium channel also plays a role [14]. Hyperkalemia has been reported to cause fatal cardiac dysrhythmia. It is also closely linked with increased mortality. Recently, studies have shown that it can cause renal tubular acidosis, and plays a role in peripheral neuropathy in patients with CKD [15]. 

To the best of our knowledge, this is the first study from this region that compares the effect of NSAIDs on potassium level in both diabetic and non-diabetic population. However, since it was a case-control study, a definite association could not be confirmed. Secondly, since the study was conducted in a single institute, the sample size was less diverse. 

## Conclusions

In this study, patients with continuous use of NSAIDs had higher levels of serum potassium compared to patients without continuous use of NSAIDs. This difference was even higher in diabetic patients. Care should be taken while prescribing patients with NSAIDs and continuous monitoring of potassium levels is needed in case of long-term use of NSAIDs.
